# Characterization of the interactions between inhibitor-1 and recombinant PP1 by NMR spectroscopy

**DOI:** 10.1038/s41598-017-18383-x

**Published:** 2018-01-08

**Authors:** Chu-Ting Liang, Yu-Shan Lin, Yi-Choang Huang, Hsien-Lu Huang, Jia-Qian Yang, Tsung-Hsien Wu, Chi-Fon Chang, Shing-Jong Huang, Hsien-Bin Huang, Ta-Hsien Lin

**Affiliations:** 1 Basic Research Division, Medical Research Department, Taipei Veterans General Hospital, Taipei, 11217 Taiwan; 2 Department of Life Sciences and Institute of Genome Sciences, National Yang-Ming University, Taipei, 11221 Taiwan; 3 Department of Life Science, National Chung Cheng University, Chia-Yi, 62102 Taiwan; 4 Institute of Biochemistry and Molecular Biology, National Yang-Ming University, Taipei, 11221 Taiwan; 5 Department of Nutrition and Health Science, Fooyin University, Kaohsiung, 83102 Taiwan; 6 Genomics Research Center, Academia Sinica, Taipei, 11529 Taiwan; 7 Instrumentation Center, National Taiwan University, Taipei, 10617 Taiwan

## Abstract

Inhibitor-1 is converted into a potent inhibitor of native protein phosphatase-1 (PP1) when Thr35 is phosphorylated by cAMP-dependent protein kinase (PKA). However, PKA-phosphorylated form of inhibitor-1 displayed a weak activity in inhibition of recombinant PP1. The mechanism for the impaired activity of PKA-phosphorylated inhibitor-1 toward inhibition of recombinant PP1 remained elusive. By using NMR spectroscopy in combination with site-directed mutagenesis and inhibitory assay, we found that the interaction between recombinant PP1 and the consensus PP1-binding motif of PKA-thiophosphorylated form of inhibitor-1 was unexpectedly weak. Unlike binding to native PP1, the subdomains 1 (residues around and including the phosphorylated Thr35) and 2 (the consensus PP1-binding motif) of PKA-thiophosphorylated form of inhibitor-1 do not exhibit a synergistic effect in inhibition of recombinant PP1. This finding implied that a slight structural discrepancy exists between native and recombinant PP1, resulting in PKA-thiophosphorylated form of inhibitor-1 displaying a different affinity to native and recombinant enzyme.

## Introduction

**Figure 1:**
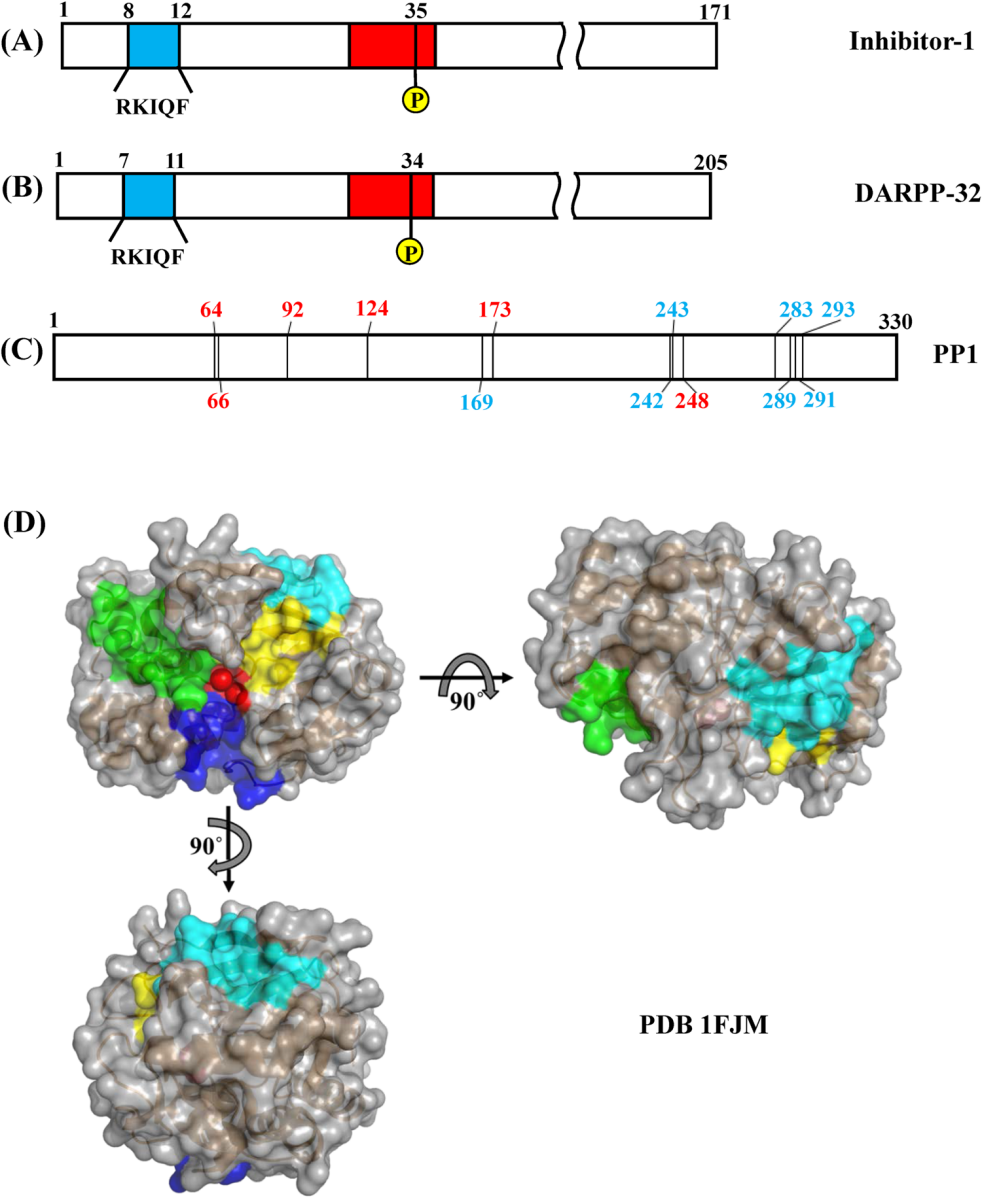
A schematic representation of the primary structures of (**A**) inhibitor-1, (**B**) DARPP32 and (**C**) PP1. Subdomain 1 (red) of inhibitor-1 is a region containing the residues close to and including phosphorylation site and subdomain 2 (cyan) of inhibitor-1 consists of a consensus PP1-binding motif, RVXF. Thr35 and Thr34 are the phosphorylation sites of inhibitor-1 and DARPP32, respectively. Residues of PP1 involved in the binding to manganese ions^12^ and RVXF motif^19^ were indicated with red and cyan numbers, respectively. (**D**) A surface representation of recombinant PP1 structure. The hydrophobic (blue), acidic (yellow) and C-terminal (green) grooves on the surface of PP1 were thought to be the possible regions for substrate binding. The catalytic site (red) is located at the interaction of the three grooves. The RVXF-binding pocket was colored in cyan.

Protein phosphatase-1 (PP1) is one of the major serine/threonine protein phosphatases in eukaryotic cells. The catalytic subunit of PP1 is associated with various regulatory subunits that target the enzyme to different sub-cellular locations and regulate the enzymatic activity toward specific substrates, accounting for PP1’s ability to regulate different cellular functions^1–4^. So far, more than 200 PP1-regulatory proteins have been identified. Most of the PP1-regulatory proteins contain one consensus PP1-binding motif, (R/K)(R/K)(V/I)X(F/W). This motif binds to the surface of PP1 far away from the catalytic site^5,6^. In addition, PP1 is also specifically inhibited by three thermostable protein inhibitors, such as inhibitor-1, DARPP-32 (dopamine- and cyclic-AMP-regulated phosphoprotein of molecular weight 32,000) and inhibitor-2. Phosphorylation of either Thr35 of inhibitor-1 or Thr34 of DARPP-32 by cAMP-dependent protein kinase (PKA) is required to convert each protein into a potent inhibitor of PP1. Inhibitor-1 and DARPP-32 share a high identity in the sequence of N-terminal region^7,8^. This region contains two subdomains that are essential in inhibition of PP1. In PKA-phosphorylated form of inhibitor-1, subdomain 1 is a region containing the residues close to and including phosphorylated Thr35, and subdomain 2 consists of a consensus PP1-binding motif located between residues 8 and 12, R_8_KIQF_12_^5,6,9–11^. Figure 1A,B and C showed a schematic illustration of the primary structures of inhibitor-1, DARPP32 and PP1, respectively. Figure 1D depicted three views of surface representation of recombinant PP1 structure^12^. The catalytic site, the consensus PP1-binding motif docking pocket and three possible substrate-binding grooves of recombinant PP1 were indicated in the figure with different colors.

PKA-phosphorylated form of inhibitor-1 is a potent inhibitor of native PP1 purified from rabbit muscles, but its inhibitory activity toward recombinant PP1 is significantly reduced^13^. Similar phenomena were also observed in PKA-phosphorylated form of DARPP-32^14,15^. In addition, PKA-phosphorylated form of inhibitor-1 and DARPP-32 can be efficiently dephosphorylated by recombinant PP1, accounting for native and recombinant PP1 displaying a subtle structural difference^13,14^. These data suggested that the interactions of PKA-phosphorylated form of inhibitor-1 with recombinant PP1 might be different from those with native PP1. The underlying mechanism for the impairment of PKA-phosphorylated form of inhibitor-1 and DARPP-32 in inhibition of recombinant PP1 remained unknown. In this study, we applied NMR titrations to characterize the interactions between PKA-thiophosphorylated form of inhibitor-1 (and inhibitor-1) and recombinant PP1. Our NMR data suggested that residues in the consensus PP1-binding motif (subdomain 2) of both non-phosphorylated and PKA-thiophosphorylated form of inhibitor-1 did not strongly bind to recombinant PP1. The results obtained from site-directed mutagenesis and inhibitory assay further supported the NMR observations. These findings may account for the reduced activity of PKA-phosphorylated form of inhibitor-1 toward inhibition of recombinant PP1.

## Results

### Interactions between non-phosphorylated form of inhibitor-1 and recombinant PP1

**Figure 2:**
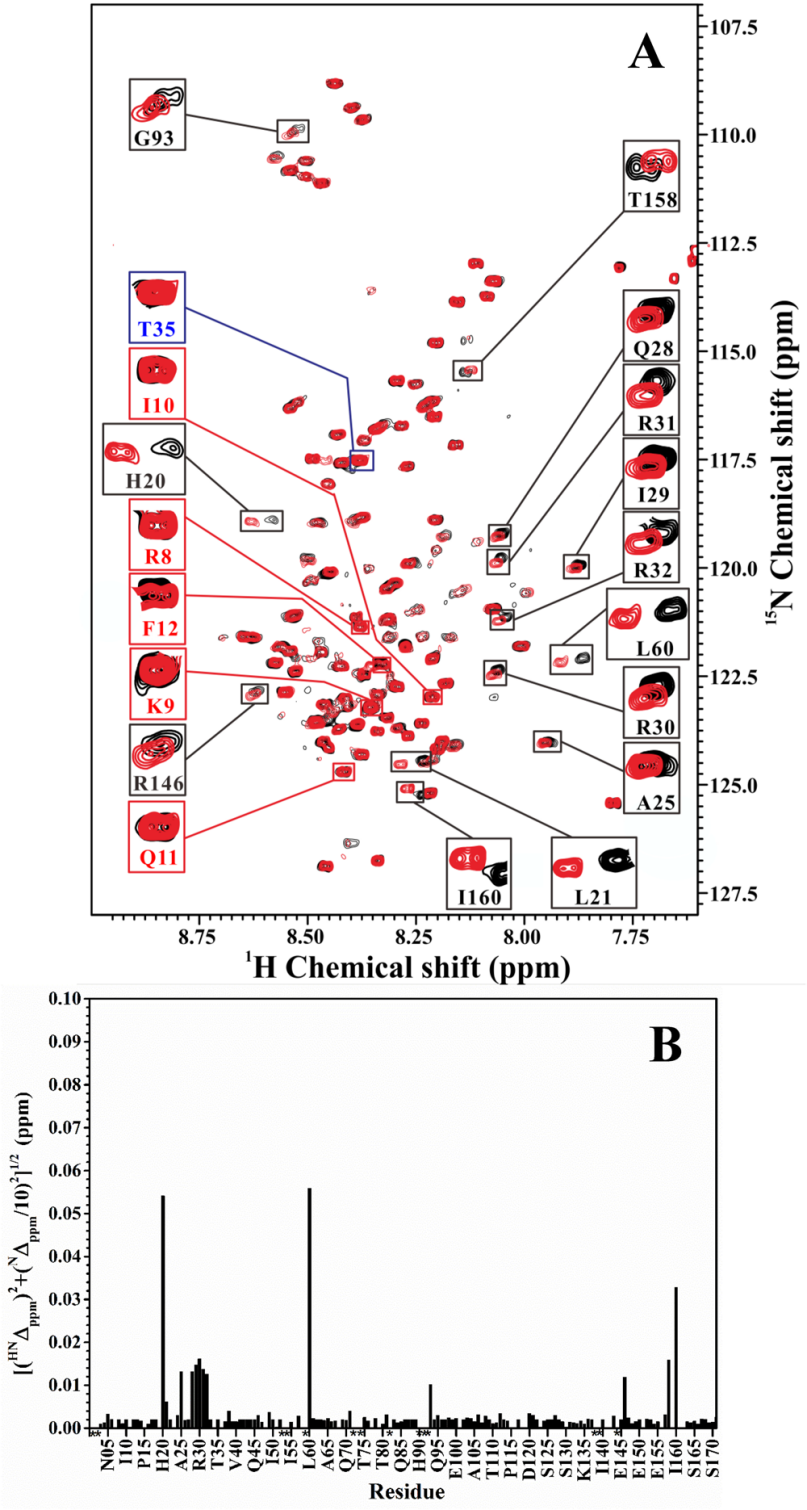
(**A**) Overlay of the two-dimensional ^1^H-^15^N-HSQC spectra of non-phosphorylated form of ^15^N-labeled inhibitor-1 (8.0 μM) in the absence (black) and presence (red) of unlabeled recombinant PP1 (8.0 μM). Blue and black boxes highlight the resonance peaks of PKA-phosphorylation site, Thr35, and residues with chemical shift perturbations, respectively. Red boxes highlight the resonance peaks of residues in the consensus PP1-binding motif. (**B**) The amide chemical shift differences between the two spectra shown in (**A**). The weighted chemical shift differences ([(^HN^Δ_ppm_)^2^ + (^N^Δ_ppm_/10)^2^]^1/2^) are plotted as a function of residue number. ^HN^Δ_ppm_ and ^N^Δ_ppm_ are the ^1^H^N^ and ^15^N chemical shift differences between non-phosphorylated form of inhibitor-1 in the absence and presence of recombinant PP1, respectively. Overlapped or missing resonance peaks are marked with star symbol.

We first characterized the interactions between non-phosphorylated form of inhibitor-1 and recombinant PP1 by using NMR spectroscopy. Two-dimensional ^1^H-^15^N-HSQC experiment was carried out on the non-phosphorylated form of ^15^N-labeled inhibitor-1 (8 μM) in the absence and presence of an equal concentration of unlabeled recombinant PP1 (8 μM). The results are shown in Fig. 2A. At first glance, it seemed that the nitrogen and amide proton cross-peaks of non-phosphorylated form of inhibitor-1 displayed no chemical shift perturbation after addition of unlabeled recombinant PP1. After expansion of the spectra, it can be seen that several nitrogen and amide proton cross-peaks of non-phosphorylated form of inhibitor-1 displayed chemical shift perturbations after addition of unlabeled recombinant PP1. Figure 2B shows the backbone amide chemical shift differences between non-phosphorylated form of inhibitor-1 in the absence and presence of recombinant PP1. These resonance peaks (expanded in black boxes of Fig. 2A) were assigned (His20, Leu21, Ala25, Gln28, Ile29, Arg30, Arg31, Arg32, Leu60, Gly93, Arg146, Thr158 and Ile160) according to the previous backbone resonance assignments of non-phosphorylated form of inhibitor-1^16^. Among these residues, His20, Leu21, Ala25, Gln28, Ile29, Arg30, Arg31 and Arg32 are located around the PKA-phosphorylation site, Thr35, in the primary sequence. The intensities of these resonance peaks were also examined. Some of these residues (Leu21, Gln28, Ile29, Arg30, Arg31, Arg32,) exhibited small intensity drop (approximate 12~29% off) after addition of unlabeled recombinant PP1. No intensity drop was observed for the phosphorylation site, Thr35 (resonance peak expanded in blue box of Fig. 2A), suggesting that it was not involved in the interaction between non-phosphorylated form of inhibitor-1 and recombinant PP1. According to the NMR data shown in Fig. 2, we inferred that non-phosphorylated form of inhibitor-1 would interact with recombinant PP1 through the residues which displayed chemical shift perturbations after addition of unlabeled recombinant PP1, but was not bound in a strong interaction manner. Those residues located around the phosphorylation site are likely to correspond to or overlap with the residues in subdomain 1 of PKA-phosphorylated form of inhibitor-1 required for inhibition of native PP1.

The resonance peaks expanded in the red boxes of Fig. 2A are the residues in the consensus PP1-binding motif, R_8_KIQF_12_ (subdomain 2 of PKA-phosphorylated form of inhibitor-1 required for inhibition of native PP1). These residues displayed no chemical shift perturbations after addition of unlabeled recombinant PP1 (resonance peak expanded in red boxes of Fig. 2A). No significant intensity drop was observed for these residues after addition of unlabeled recombinant PP1, except I10 (approximate 16% off). These results suggested that the residues in the consensus PP1-binding motif might interact very weakly with recombinant PP1 when inhibitor-1 was non-phosphorylated.

### Interactions between PKA-thiophosphorylated form of inhibitor-1 and recombinant PP1

**Figure 3:**
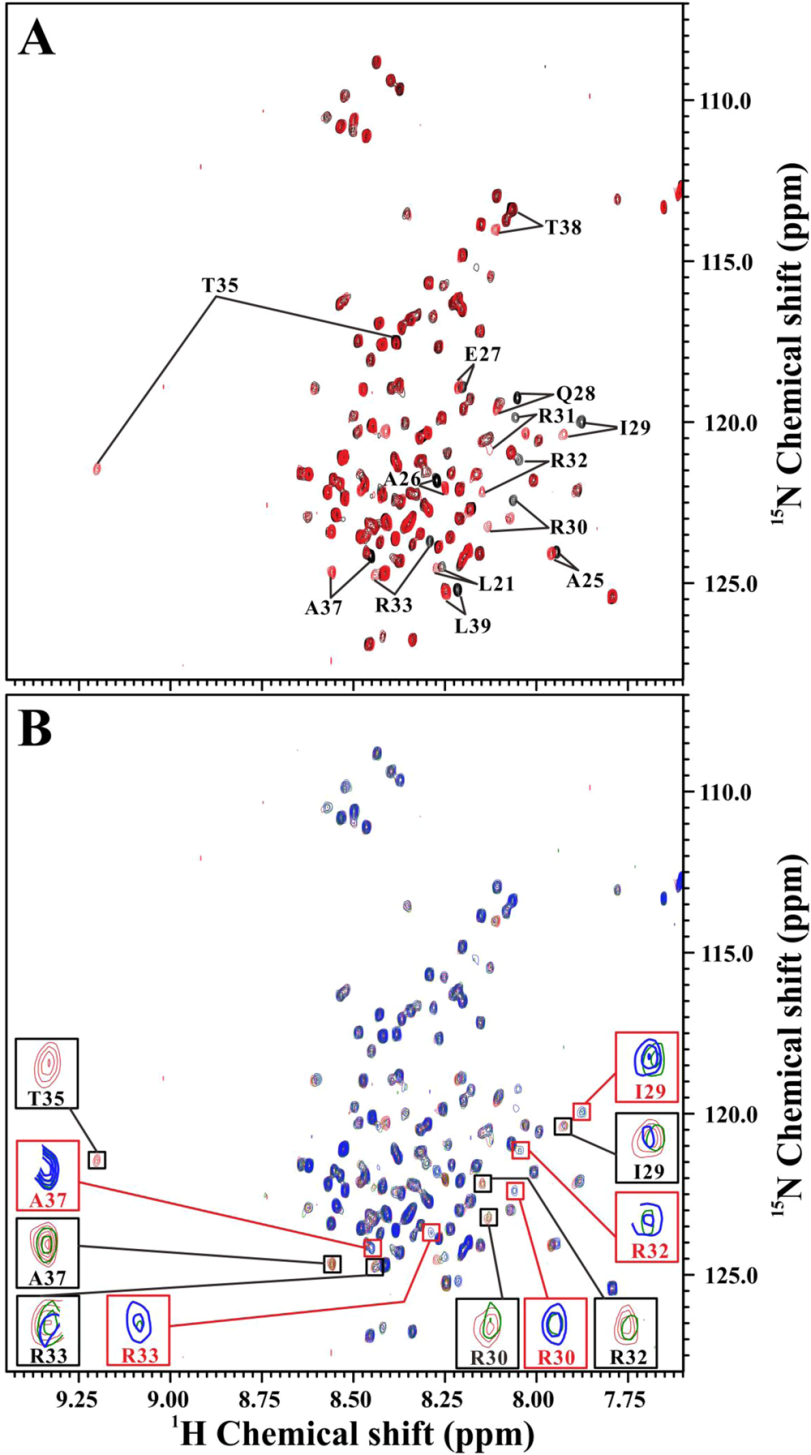
The effect of PKA-thiophosphorylation on the overall structure of inhibitor-1 and dephosphorylation of PKA-thiophosphorylated form of inhibitor-1. (**A**) Overlay of the two-dimensional ^1^H-^15^N-HSQC spectra of PKA-thiophosphorylated (red) and non-phosphorylated (black) forms of ^15^N-labeled inhibitor-1. For clarity, only the residues with significant chemical shift changes induced by thiophosphorylation of Thr35 were indicated. (**B**) Overlay of the two-dimensional ^1^H-^15^N-HSQC spectra of PKA-thiophosphorylated form of ^15^N-labeled inhibitor-1 (40 μM) acquired at different time points after addition of unlabeled recombinant PP1 (8.5 μM). The spectrum acquired without addition of unlabeled recombinant PP1 was colored in red. The spectra acquired at 32 and 52 minutes after addition of unlabeled recombinant PP1 were colored in green and blue, respectively. Black and red boxes highlight the resonance peaks of residues of PKA-thiophosphorylated and non-phosphorylated forms of inhibitor-1, respectively. For clarity, we only highlighted resonance peaks of I29, R30, R32, R33, T35 and A37.

We next investigated the effect of PKA-phosphorylation on the interactions of inhibitor-1 with recombinant PP1. The recombinant PP1 would dephosphorylate PKA-phosphorylated form of inhibitor-1 rapidly. In order to observe the interactions between PKA-phosphorylated form of inhibitor-1 and recombinant PP1, PKA-thiophosphorylated form of inhibitor-1 was used to reduce the rate of dephosphorylation during NMR titrations. First of all, we checked the effect of PKA-thiophosphorylation on the overall structure of inhibitor-1. Figure 3A shows the result of resonance assignment of PKA-thiophosphorylated form of inhibitor-1. Previously, we have shown that the effect of PKA-phosphorylation on the overall structure of inhibitor-1 is insignificant^16^. The spectral patterns shown in Fig. 3A are very similar to those obtained from PKA-phosphorylated and non-phosphorylated forms of inhibitor-1 as described^16^, suggesting that the effect of thiophosphorylation on the overall structure of inhibitor-1 is insignificant as well.

To obtain an optimal condition for observing the interactions between PKA-thiophosphorylated form of inhibitor-1 and recombinant PP1, we examined the rate of dephosphorylation of PKA-thiophosphorylated form of inhibitor-1 catalyzed by recombinant PP1. A time-dependent series of two-dimensional ^1^H-^15^N-HSQC experiments were immediately performed on PKA-thiophosphorylated form of ^15^N-labeled inhibitor-1 after addition of unlabeled recombinant PP1. By varying the concentrations of PKA-thiophosphorylated form of inhibitor-1 and recombinant PP1, we obtained an optimal condition under which the overall dephosphorylation process of PKA-thiophosphorylated form of inhibitor-1 can be observed by NMR spectroscopy. Figure 3B shows the dephosphorylation process of PKA-thiophosphorylated form of ^15^N-labeled inhibitor-1 (40 μM) after addition of unlabeled recombinant PP1 (8.5 μM) monitored by two-dimensional ^1^H-^15^N-HSQC experiments. The resonance peak intensities of the residues close to the thiophosphorylated Thr35 decreased over time after addition of unlabeled recombinant PP1 (resonance peaks expanded in black boxes of Fig. 3B). Some of the resonance peaks appeared after addition of unlabeled recombinant PP1. The intensities of these resonance peaks increased over time (resonance peaks expanded in red boxes of Fig. 3B). These resonance peaks were assigned to the residues of non-phosphorylated form of inhibitor-1 according to the previous resonance assignments^16^. These phenomena indicated that PKA-thiophosphorylated form of inhibitor-1 was dephosphorylated by recombinant PP1. For the thiophosphorylated Thr35, its resonance peak disappeared in the first two-dimensional ^1^H-^15^N-HSQC spectrum acquired at 22 minutes after addition of unlabeled recombinant PP1 (Supplementary data Fig. S1). Although PKA-thiophosphorylated form of inhibitor-1 was employed to reduce the rate of dephosphorylation, it remained to be dephosphorylated by recombinant PP1 at a moderate rate.

**Figure 4:**
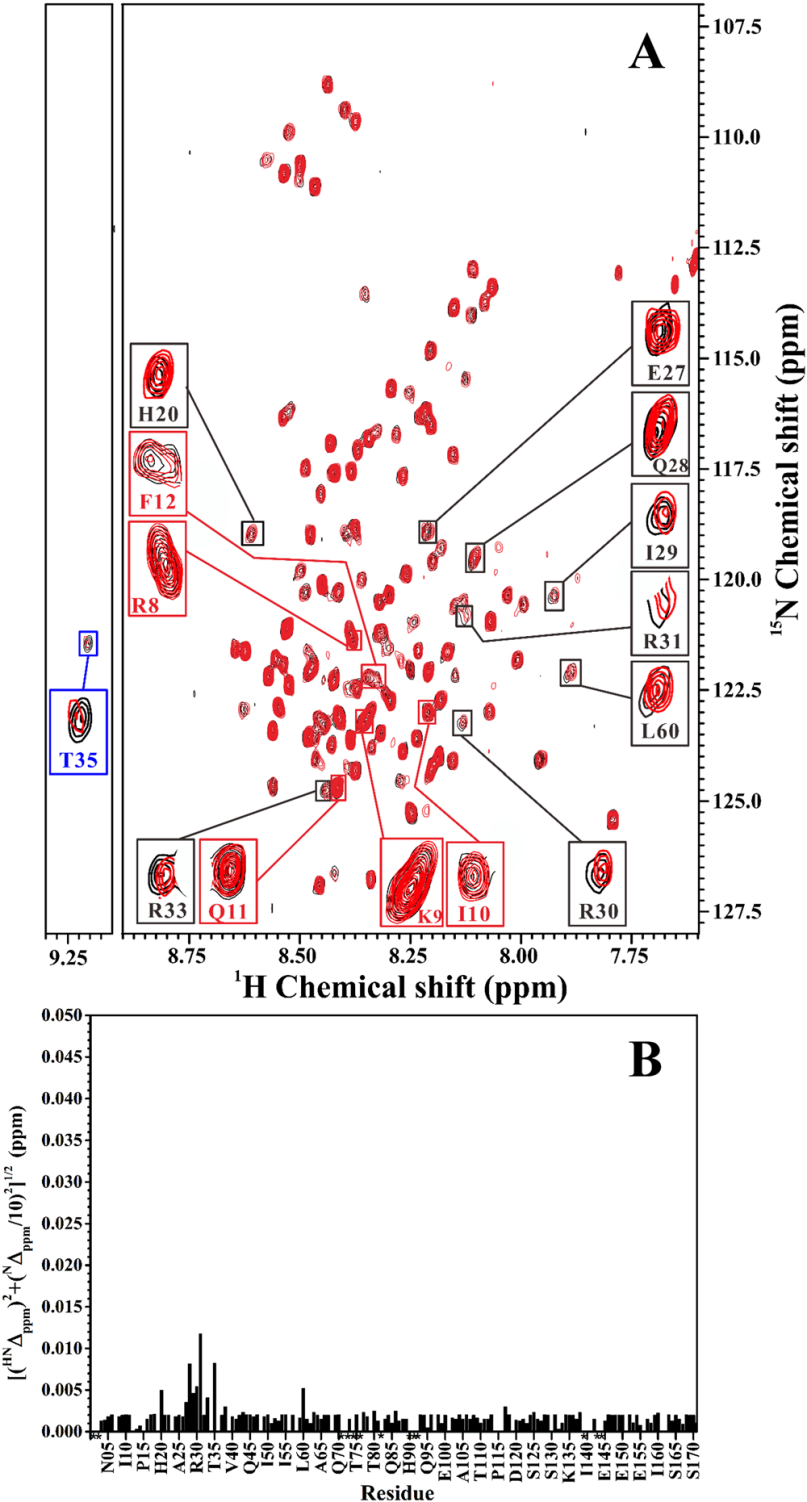
(**A**) NMR titration of the PKA-thiophosphorylated form of ^15^N-labeled inhibitor-1 (40 μM) with different concentrations of unlabeled recombinant PP1 (8.5 μM and 6.0 μM). For clarity, only the two-dimensional ^1^H-^15^N-HSQC spectra of PKA-thiophosphorylated form of ^15^N-labeled inhibitor-1 in the absence (black) and presence (red) of 6.0 μM recombinant PP1 were shown. Blue and black boxes highlight the resonance peaks of PKA-thiophosphorylation site, Thr35, and residues with slight chemical shift perturbations, respectively. Red boxes highlight the resonance peaks of residues in the consensus PP1-binding motif. (**B**) The amide chemical shift differences between the two spectra shown in (**A**). The weighted chemical shift differences ([(^HN^Δ_ppm_)^2^ + (^N^Δ_ppm_/10)^2^]^1/2^) are plotted as a function of residue number. ^HN^Δ_ppm_ and ^N^Δ_ppm_ are the ^1^H^N^ and ^15^N chemical shift differences between PKA-thiophosphorylated form of inhibitor-1 in the absence and presence of recombinant PP1., respectively. Overlapped or missing resonance peaks are marked with star symbol.

In order to observe the resonance peak of thiophosphorylated Thr35, we reduced the concentration of unlabeled recombinant PP1 (6.0 μM) to titrate PKA-thiophosphorylated form of inhibitor-1 (40 μM). Figure 4A shows the superimposed two-dimensional ^1^H-^15^N-HSQC spectra of PKA-thiophosphorylated form of ^15^N-labeled inhibitor-1 in the absence and presence of unlabeled recombinant PP1. A plot of the backbone amide chemical shift differences vs. residue number is shown in Fig. 4B. The spectral pattern shown in Fig. 4A looks similar to those shown in Fig. 3B, but the resonance peak of thiophosphorylated Thr35 can be observed after the rate of dephosphorylation was reduced. It can be seen that the resonance peak of thiophosphorylated Thr35 (resonance peak expanded in the blue box of Fig. 4A) displays a slight chemical shift perturbation and an intensity drop after addition of unlabeled recombinant PP1, indicating that thiophosphorylated Thr35 interacted with recombinant PP1. The drop in peak intensity of thiophosphorylated Thr35 should be derived from the effect of dephosphorylation. It might also be partly due to the interaction between thiophosphorylated Thr35 and recombinant PP1. The reaction of dephosphorylation is also indicative of the existence of a strong interaction between thiophosphorylated Thr35 and recombinant PP1.

Slight chemical shift perturbations are observed for some of the nitrogen and amide proton cross-peaks of PKA-thiophosphorylated form of inhibitor-1 after addition of unlabeled recombinant PP1. Resonance peaks which display slight chemical shift perturbations (His20, Glu27, Gln28, Ile29, Arg30, Arg 31, Arg 33 and Leu60) are expanded in black boxes of Fig. 4A. These results suggest that PKA-thiophosphorylated form of inhibitor-1 might interact with recombinant PP1 through these residues. We also found that residues in the consensus PP1-binding motif (subdomain 2) of PKA-thiophosphorylated form of inhibitor-1 displayed no chemical shift changes of their nitrogen and amide proton cross-peaks (resonance peaks expanded in red boxes of Fig. 4A) after addition of unlabeled recombinant PP1, suggesting that the consensus PP-1 binding motif might not interact or weakly interacted with recombinant PP1 when inhibitor-1 was thiophosphorylated by PKA. It has to be cautious to interpret the interaction strength between these residues of PKA-thiophosphorylated form of inhibitor-1 and recombinant PP1 solely based on their chemical shift perturbations, in particular when they displayed slight or no perturbation. Since the concentration of recombinant PP1 (6.0 μM or 8.5 μM) is relatively low as compared with that of PKA-thiophosphorylated form of inhibitor-1 (40 μM) and the molecular weight of PP1 is high (M.W. ~38 kDa), the spectral patterns might look similar to those shown in Fig. 4A in case of an intermediate or a strong interaction existing between these residues and recombinant PP1. The alteration of resonance peak intensity (or linewidth) needs to be taken into account as well. However, due to the low solubility of recombinant PP1 and its ability to catalyze the dephosphorylation of PKA-thiophosphorylated form of inhibitor-1, the highest concentration of recombinant PP1 used for NMR titrations was limited to 8.5 μM. Under this condition, it is difficult to analyze the change of resonance peak intensity (or linewidth) accurately. To confirm the results obtained from the NMR analysis, we next performed site-directed mutagenesis on the consensus PP1-binding motif and analyzed the inhibitory potency of PKA-thiophosphorylated form of inhibitor-1 mutant toward recombinant PP1.

### Inhibitory activities of PKA-thiophosphorylated form of inhibitor-1 and inhibitor-1[I10A;F12A]

**Figure 5:**
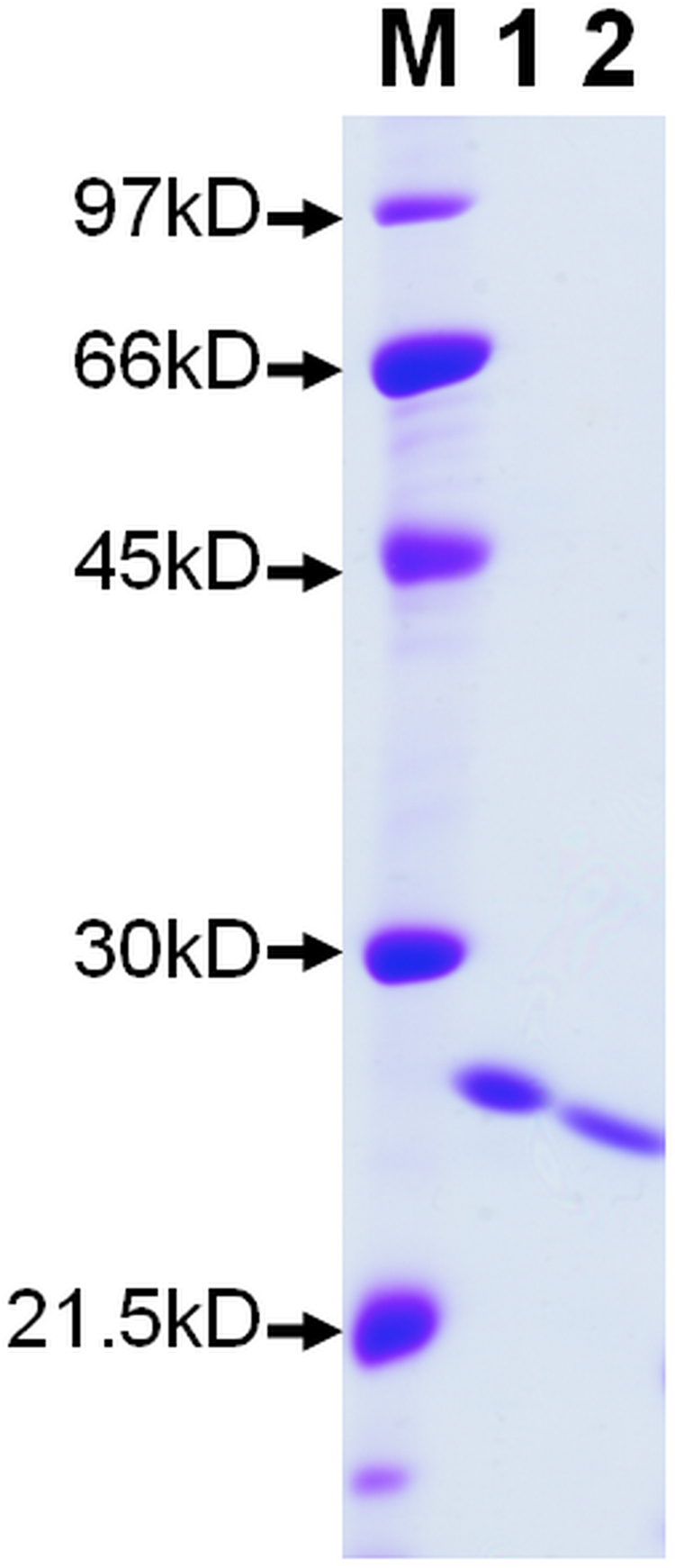
SDS-PAGE of inhibitor-1 and inhibitor-1[I10A;F12A]. Aliquots of inhibitor-1 (2 μg) and inhibitor-1[I10A;F12A] (2 μg) were separated on SDS-PAGE (12.5%), and stained with Coomassie Brilliant Blue. Lane M is the molecular weight markers. Lane 1 and 2 represent inhibitor-1 and inhibitor-1[I10A;F12A], respectively.

**Figure 6:**
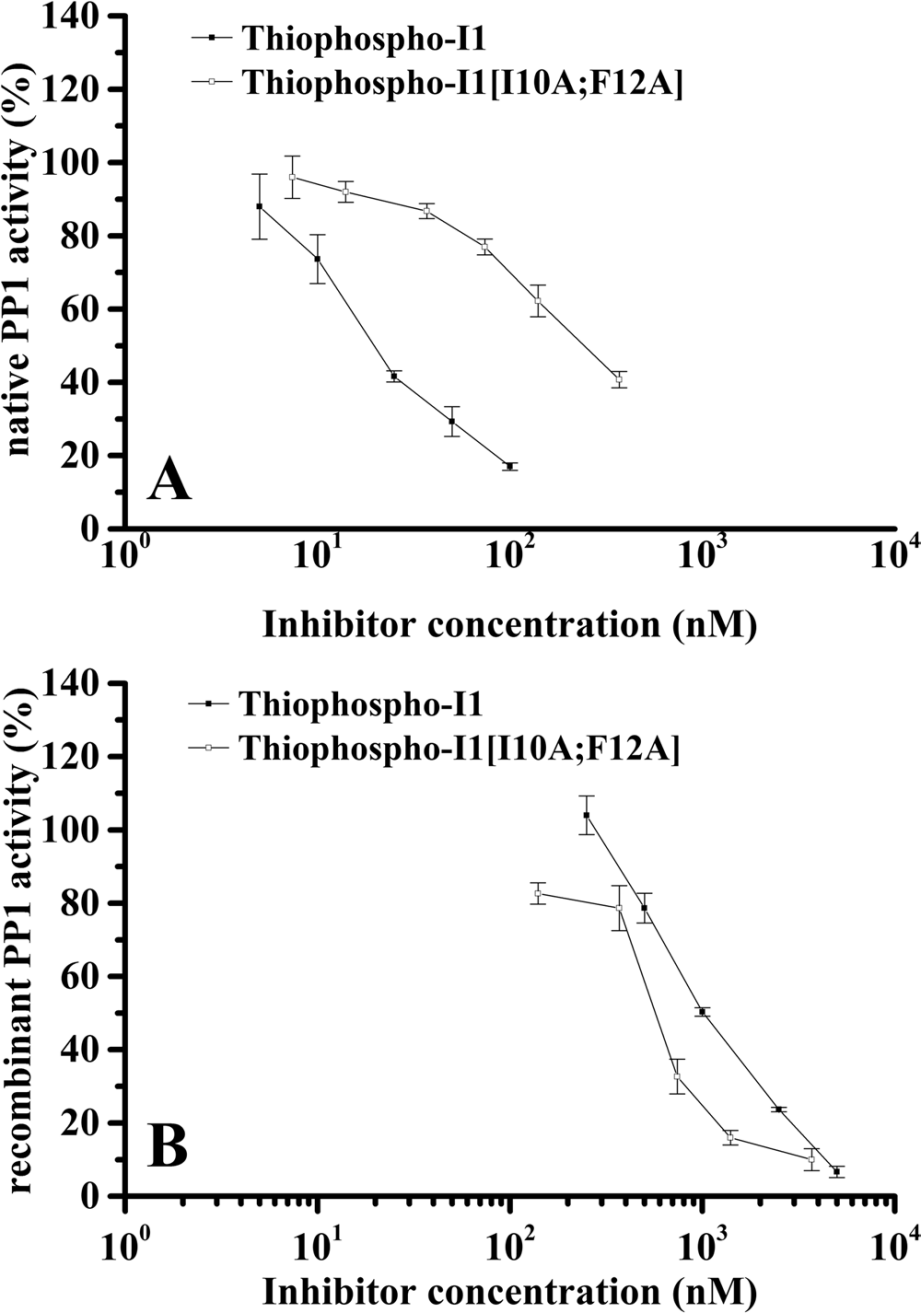
The inhibitory assay of PP1. PP1 was assayed using 10 mM [^32^P]phosphorylase a as substrate. PKA-thiophosphorylated form of inhibitor-1 (filled square) and PKA-thiophosphorylated form of inhibitor-1[I10A;F12A] (open square) are present in the indicated concentrations. Phosphatase activity is expressed as percent of the activity measured in the absence of PKA-thiophosphorylated form of inhibitor-1. (**A**) and (**B**) represent the inhibitory curves of native and recombinant PP1 by PKA-thiophosphorylated form of inhibitor-1 and inhibitor-1[I10A;F12A], respectively.

According to the result of chemical shift perturbation analysis, we obtained that the consensus PP1-binding motif of PKA-thiophosphorylated form of inhibitor-1 might not interact or interacted very weakly with recombinant PP1. If this is true, then mutation of the residues in the consensus PP1-binding motif will have no significant effect on the inhibitory activity of PKA-thiophosphorylated form of inhibitor-1 toward recombinant PP1. To demonstrate this hypothesis, we prepared inhibitor-1[I10A;F12A] and its PKA-thiophosphorylated form for inhibitory assay. Figure 5 shows the result of SDS-PAGE analysis of inhibitor-1[I10A;F12A] and its PKA-thiophosphorylated form in which the purity of both proteins after three steps of purification is more than 95% homogeneity. We first measured the inhibitory activities of PKA-thiophosphorylated forms of inhibitor-1 and inhibitor-1[I10A;F12A] toward native PP1. The results are shown in Fig. 6A. The values of IC_50_ for inhibition of native PP1 by PKA-thiophosphorylated forms of inhibitor-1 and inhibitor-1[I10A;F12A] were 19 ± 1 nM (n = 3) and 240 ± 39 nM (n = 4) (Table 1), respectively. The IC_50_ value of PKA-thiophosphorylated form of inhibitor-1[I10A/F12A] is approximately 12.6-fold higher than that of PKA-thiophosphorylated form of inhibitor-1 for inhibition of native PP1, indicating that replacement of critical residues in the consensus PP1-binding motif, R_8_KIQF_12_, significantly reduces the inhibitory activity of PKA-thiophosphorylated form of inhibitor-1 toward native PP1. This result may suggest that residues in the region of consensus PP1-binding motif strongly interacted with native PP1. We next performed the inhibitory assay of recombinant PP1. The results are shown in Fig. 6B. The values of IC_50_ for inhibition of recombinant PP1 by PKA-thiophosphorylated forms of inhibitor-1 and inhibitor-1[I10A;F12A] are 980 ± 41 nM (n = 3) and 553 ± 23 nM (n = 3) (Table 1), respectively. The IC_50_ ratio of PKA-thiophosphorylated form of inhibitor-1[I10A;F12A] to PKA-thiophosphorylated form of inhibitor-1 is close to 0.6, indicating that replacement of critical residues in the consensus PP1-binding motif did not significantly impair the inhibitory activity of PKA-thiophosphorylated form of inhibitor-1 toward recombinant PP1. This finding suggests that the interaction between the consensus PP1-binding motif and recombinant PP1 is not strong, confirming our NMR analysis. Furthermore, the IC_50_ value for inhibition of recombinant PP1 (553 ± 23 nM) by PKA-thiophosphorylated form of inhibitor-1[I10A;F12A] may also reflect the binding strength of thiophosphorylated Thr35 and residues around it (or subdomain 1) to recombinant PP1, if there is no occurrence of dephosphorylation at Thr35. It seems that this interaction is not very strong. However, the reaction of dephosphorylation suggests that there is a strong interaction between thiophosphorylated Thr35 and recombinant PP1. This phenomenon might be due to the difference in the rates of dephosphorylation and dissociation of PKA-thiophosphorylated form of inhibitor-1 from recombinant PP1 (*k*_off_). If the rate of dephosphorylation is much faster than that of dissociation, even the rate of dissociation is very slow, the strong interaction between thiophosphorylated Thr35 and recombinant PP1 would be difficult to be observed.

**Table 1:** IC_50_ of PP1 inhibited by PKA-thiophosphorylated forms of inhibitor-1 and inhibitor-1[I10A;F12A].

Inhibitor	Native PP1	Recombinant PP1
	IC_50_ (nM)	
PKA-thiophosphorylated form of inhibitor-1	19 ± 1	980 ± 41
PKA-thiophosphorylated form of inhibitor-1[I10A;F12A]	240 ± 39	553 ± 23

IC_50_ values were determined from results shown in Fig. 6A and B. Results are expressed as mean ± S.D.

## Discussion

In the previous study, we have shown that the values of IC_50_ for inhibition of native PP1 by non-phosphorylated and PKA-phosphorylated forms of inhibitor-1 were 65 ± 8 μM and 6.0 ± 0.9 nM, respectively^17^. The potency of inhibitor-1 for inhibition of native PP1 is remarkably increased after PKA-phosphorylation. In general, the value of IC_50_ is related to the value of *K*_d_ (dissociation constant) which is a measurement of binding affinity. The IC_50_ values of non-phosphorylated and PKA-phosphorylated forms of inhibitor-1 implied that the *K*_d_ value for the binding of non-phosphorylated form of inhibitor-1 to native PP1 should be much higher than that for the binding of PKA-phosphorylated form of inhibitor-1 to native PP1. PKA-phosphorylation on Thr35 increases the affinity of inhibitor-1 binding to native PP1.

If the structural conformation between native and recombinant PP1 is the same, there would be no difference in their affinities with non-phosphorylated form of inhibitor-1. We would expect to observe weak interactions between recombinant PP1 and non-phosphorylated inhibitor-1 according the value of IC_50_ for inhibition of native PP1 by non-phosphorylated inhibitor-1 (65 ± 8 μM). In this study, our NMR results suggest that non-phosphorylated form of inhibitor-1 weakly binds to recombinant PP1 through the consensus PP1-binding motif and the residues located around, but not including, the phosphorylation site, Thr35 (Fig. 2A). These binding modules may also be a model for the interaction between native PP1 and non-phosphorylated form of inhibitor-1.

The IC_50_ value for inhibition of native PP1 by PKA-thiophosphorylated form of inhibitor-1 is 19 ± 12 nM (Table 1). This may suggest that PKA-thiophosphorylated form of inhibitor-1 strongly binds to native PP1. Furthermore, our previous studies have demonstrated that phosphorylation at Thr35 by PKA had no significant effect on the overall structure of inhibitor-1^16^. Similar phenomenon was also observed in PKA-thiophosphorylated form of inhibitor-1, as revealed by the 2D HSQC spectra (Fig. 3A). From a structural perspective, we may infer that the interactions of PKA-thiophosphorylated (or PKA-phosphorylated) inhibitor-1 with native PP1 should be similar to those of non-phosphorylated inhibitor-1 with native PP1, except the interaction occurred at the PKA-thiophosphorylation (or PKA-phosphorylation) site. This suggests that the strong interaction between PKA-thiophosphorylated (or PKA-phosphorylated) form of inhibitor-1 and native PP1 is mainly contributed by the binding of thiophosphorylated Thr35 and residues around it (subdomain 1) to native PP1.

Based on the discussion above, we speculate that subdomain 2 should bind weakly to native PP1. However, this is inconsistent with the result obtained from the site-directed mutagenesis. It suggests that a strong interaction exists between subdomain 2 and native PP1. Mutations at subdomain 2 significantly reduce the inhibition of native PP1 by PKA-thiophosphorylated form of inhibitor-1 (Fig. 6A, Table 1). A possible explanation for this phenomenon is that neither one of these two subdomains alone interact very strongly with native PP1. Subdomain 1 and 2 of PKA-thiophosphorylated form of inhibitor-1 synergistically bind to native PP1.

Under the PKA-thiophosphorylated stage of inhibitor-1, the IC_50_ value for inhibition of recombinant PP1 (980 ± 41 nM) is increased about 51-fold with respect to that for inhibition of native PP1 (19 ± 12 nM), suggesting that the overall strength of binding of PKA-thiophosphorylated form of inhibitor-1 to recombinant PP1 is weaker than that to native PP1. This might result from the difference in the strength of binding of subdomain 2 to recombinant and native PP1. The results of NMR analysis (Fig. 4A) and inhibitory assay (Fig. 6B, Table 1) suggest that the interaction between subdomain 2 and recombinant PP1 is weak, however, subdomain 2 binds strongly to native PP1 (discussed in the previous paragraph). Unlike the interaction between PKA-thiophosphorylated form of inhibitor-1 and native PP1, the interaction between PKA-thiophosphorylated form of inhibitor-1 and recombinant PP1 mainly comes from the binding of residues which correspond to or overlap with the residues in subdomain 1 to recombinant PP1. In other words, the synergistic binding of subdomain 1 and 2 to recombinant PP1 might be impaired. PKA-thiophosphorylated form of inhibitor-1 is a potent inhibitor for native PP1, but not for recombinant PP1. This alternation might arise from the impairment of synergistic binding which would reduce the activity of PKA-phosphorylated form of inhibitor-1 in inhibition of recombinant PP1. It might also partly arise from the dephosphorylation reaction catalyzed by recombinant PP1.

**Figure 7:**
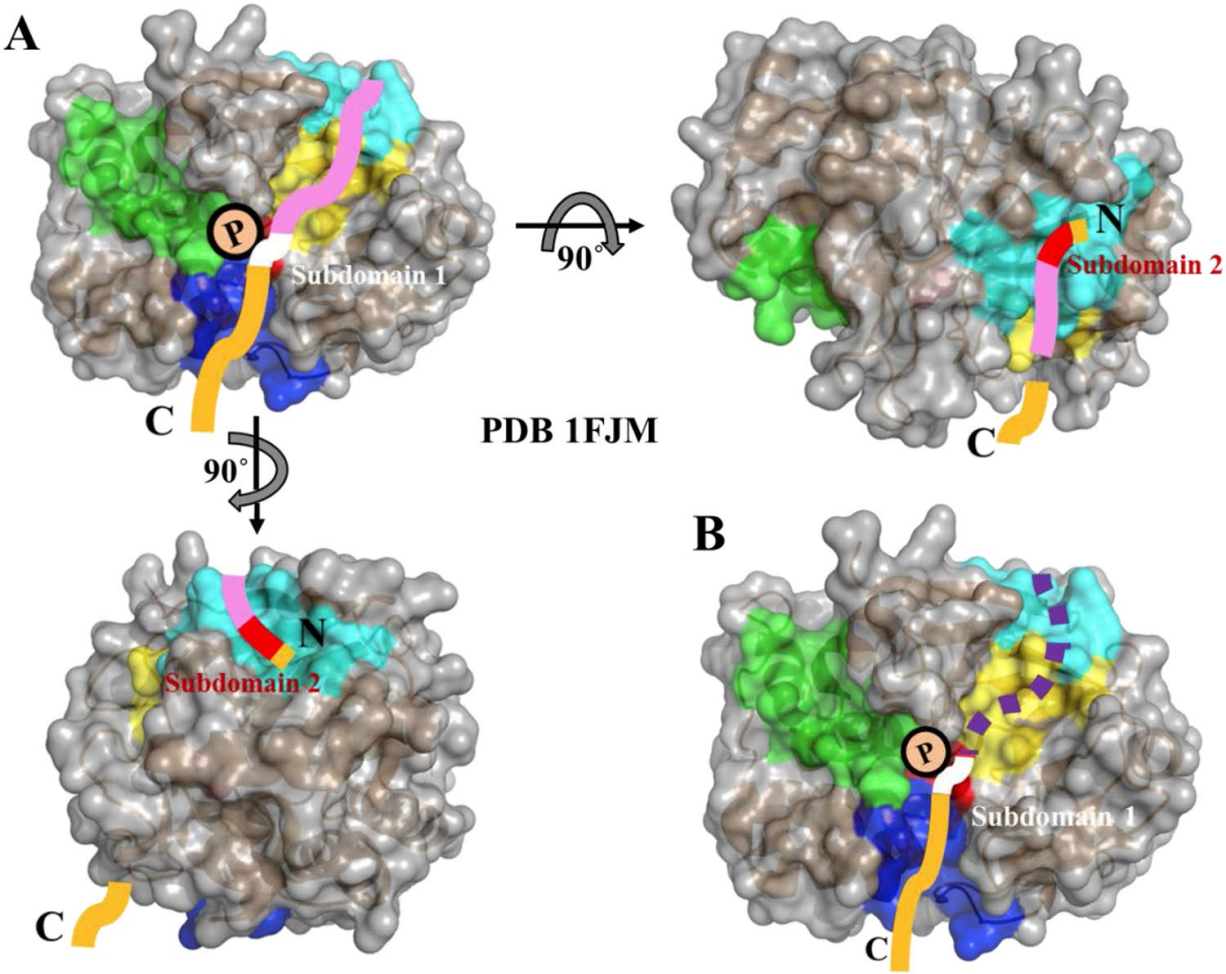
(**A**) A possible mode of binding between PKA-phosphorylated form of inhibitor-1 and recombinant PP1. This model is adapted from ref.^19^. If there is no structural difference between recombinant and native PP1, this model would be suitable for depicting the structure of PKA-phosphorylated form of inhibitor-1 in complex with native PP1. The structure of recombinant PP1 is shown in grey surface representation. The colored regions on recombinant PP1 are catalytic site (red), RVXF-binding pocket (cyan), hydrophobic (blue), acidic (yellow) and C-terminal (green) grooves. PKA-thiophosphorylated form of inhibitor-1 is shown in colored bold line (red, white, light purple and orange). PKA-thiophosphorylated form of inhibitor-1 interacts with RVXF-binding pocket (cyan), acidic groove (yellow) and catalytic site (red) of PP1 through the region from subdomain 2 (red) to subdomain 1 (white), including the region between these two domains (light purple). The region which did not interact with PP1 was colored in orange. (**B**) A hypothetic model of PKA-phosphorylated form of inhibitor-1 docking on recombinant PP1. The region (dashed line colored in purple) from the n-terminus to subdomain 1 weakly binds to the RVXF-binding pocket (cyan) and acidic groove (yellow) of recombinant PP1.

Two structural models have been proposed to illustrate the interactions between inhibitor-1 and PP1. By using the knowledge of the X-ray crystal structure of recombinant PP1 and the RVXF-binding pocket on recombinant PP1, Barford *et al*. predicted a complex structure of the N-terminal domain (residues 8 to 38) of PKA-phosphorylated form of inhibitor-1 bound to PP1^18^. Terrak *et al*. also proposed a model based on the crystal structure of recombinant PP1 in complex with MYPT1 to describe how PKA-phosphorylated form of inhibitor-1 interacted with PP1^19^. Both of these two models suggested that residues in the region between RVXF motif and phosphorylation site of inhibitor-1 might dock onto the acidic groove of recombinant PP1. Figure 7A shows a surface representation of recombinant PP1 in complex with PKA-phosphorylated form of inhibitor-1. This figure is adapted from the model proposed by Terrak *et al*.^19^. PKA-phosphorylated form of inhibitor-1 follows the path from RVXF-binding pocket to catalytic site through the acidic groove to interact with recombinant PP1. This mode of binding is the best possible way for the docking of subdomain 1 and 2 onto recombinant PP1. Assuming that there is no structural difference between native and recombinant PP1. This model can also be used for describing the mode of binding between PKA-phosphorylated form of inhibitor-1 and native PP1.

From a structural point of view, the difference in the interaction between native and recombinant PP1 with PKA-thiophosphorylated form of inhibitor-1 might result from a slightly structural difference between native and recombinant PP1^13–15^. It is possible that the structural integrity of the docking grooves for PKA-thiophosphorylated form of inhibitor-1 is impaired when PP1 was overexpressed in *E. coli*. The relative position and/or orientation between catalytic site and RVXF-binding pocket might have been altered on recombinant PP1. These factors might only affect the strength of interaction between PKA-thiophosphorylated form of inhibitor-1 and recombinant PP1, but not the mode of binding. Based on the results obtained from this study, we propose a model to depict how PKA-phosphorylated form of inhibitor-1 dock onto recombinant PP1. This hypothetical model is shown in Fig. 7B. Subdomain 1 of PKA-phosphorylated form of inhibitor-1 would strongly bind to recombinant PP1, however, subdomain 2 is weakly bound to recombinant PP1. We speculate that the region from the n-terminus to subdomain 1 of PKA-phosphorylated form of inhibitor-1, but not including subdomain 1, loosely docks onto the RVXF-binding pocket and acidic groove of recombinant PP1 through subdomain 2. The binding of this region to recombinant PP1 is more dynamic than that to native PP1, but the modes of binding for this region to recombinant and native PP1 are similar. The X-ray crystal structures of recombinant PP1 complexed with various regulatory proteins or with the peptide indicated that the consensus PP1-binding motif of different PP1-regulatory proteins docked onto the molecular surface of recombinant PP1 at the same docking groove^6,19–21^, suggesting that the structural integrity of RVXF-binding pocket might be unimpaired on recombinant PP1. Some of PP1-regulatory proteins adopt different types of binding mode to recombinant PP1. Unlike PKA-phosphorylated form of inhibitor-1 and DARPP-32, the binding affinity of these PP1-regulatory proteins to recombinant PP1 might not be affected by the structural difference between native and recombinant PP1.

Phosphorylated Thr35 strongly binds to the catalytic site of recombinant PP1, but it would be dephosphorylated by recombinant PP1. A possible explanation for this phenomenon is that the two chelated metal ions at the catalytic site of recombinant PP1 are different from those at the catalytic site of native PP1. PP1 is a metalloenzyme. Two metal ions located at the active site are required for the catalytic activity of PP1. For the native PP1, the two metal ions are zinc and iron^22^. Both ions tightly bind to the catalytic site of native PP1 and show a resistance to inhibition by EDTA. However, the two metal ions in the *E. coli*-expressed PP1 are manganese^23^. One of the manganese ions loosely binds to the catalytic site of recombinant PP1. The phosphate group of substrate is coordinated by both metal ions at the catalytic site of PP1. The presence of different metal ions at the catalytic site of PP1 might result in the significantly distinct ability toward inhibition by PKA-phosphorylated form of inhibitor-1 and DARPP-32. Both PKA-phosphorylated form of inhibitor-1 and DARPP-32 are the potent inhibitors of native PP1, but convert to excellent substrates of the *E. coli*-expressed PP1. The binding of subdomain 1 and 2 to the catalytic site and RVXF-binding site, respectively, has been changed when PKA-phosphorylated form of inhibitor-1 binds to recombinant PP1. This change might also arise from the presence of different metal ions at the catalytic site. With the exception of inhibitory activity of PKA-phosphorylated form of inhibitor-1 and DARPP-32, recombinant and native PP1 show similar function when they interact with other PP1-interacting molecules, such as inhibitor-2, organic toxins and other regulatory subunits, suggesting that the recombinant PP1 remains a good model for native PP1. So far, there is no structural information for native PP1. The structural basis for the mechanism that leads to the weak interaction between RVXF motif of PKA-phosphorylated form of inhibitor-1 and recombinant PP1 remains to be investigated.

## Methods

### Materials

ATP, dithiothreitol, HEPES, Tris base, glycine, PKA, ATP-γ-S and SDS were obtained from Sigma-Aldrich. [γ-^32^P]ATP was obtained from NEN Life Science Product. The native PP1 purified from rabbit muscles followed the methods as described^24^. The recombinant PP1 over-expressed from *E. coli* was prepared as previously described^12^. The recombinant inhibitor-1 and inhibitor-1[I10A;F12A] were prepared as previously described^17^. Phosphorylation or thiophosphorylation of inhibitor-1 by PKA followed the methods as described^14^. The recombinant inhibitor-1 labeled by stable isotopes [^13^C and ^15^N] followed the methods as described^17^. ^32^P-phosphorylase a was prepared as previously described^25^.

### Ethics Statement

The female New Zealand white rabbits were ordered from Livestock Research Institute, Council of Agriculture (Tainan, Taiwan). Before sacrifice, all the animals were maintained under a 12-h light/dark cycle at a constant temperature (22 °C) and were offered access to water and standard laboratory chow. The care, handling and experimental procedures involving rabbits were followed according to the NIH Guide for the Care and Use of Laboratory Animals (1996) and were approved by the Institute Animal Care and Use Committee (IACUC) of National Chung Cheng University.

### NMR Spectroscopy

For the study of the interactions between non-phosphorylated form of inhibitor-1 and recombinant PP1, the non-phosphorylated form of ^15^N-labeled inhibitor-1 (8.0 μM) was titrated with unlabeled recombinant PP1 (8.0 μM) at pH 6.0 (the highest concentration of recombinant PP1 in our preparation is 8.5 μM). Two-dimensional ^1^H-^15^N-HSQC spectra (2048 × 128 complex points, 1024 scans) of non-phosphorylated form of inhibitor-1 in the absence and presence of unlabeled recombinant PP1 were acquired on a 500 MHz NMR spectrometer. To monitor the process of PKA-thiophosphorylated form of inhibitor-1 dephosphorylated by recombinant PP1, two-dimensional ^1^H-^15^N-HSQC spectra of PKA-thiophosphorylated form of ^15^N-labeled inhibitor-1 (40 μM) in the presence of unlabeled recombinant PP1 (8.5 μM) at pH 6.0 were acquired sequentially. The dead time of the time-dependent experiments was approximate 12 minutes from the sample preparation to the beginning of data acquisition of the first two-dimensional ^1^H-^15^N-HSQC experiment. The time for acquisition of a two-dimensional ^1^H-^15^N-HSQC spectrum with sufficient signal to noise ratio and resolution (2048 × 64 complex points, 4 scans) on an 800 MHz NMR spectrometer was approximate 10 minutes. For the study of the interactions between PKA-thiophosphorylated form of inhibitor-1 and recombinant PP1, the PKA-thiophosphorylated form of ^15^N-labeled inhibitor-1 (40 μM) was titrated with unlabeled recombinant PP1 (6.0 μM and 8.5 μM) at pH 6.0. Backbone sequential assignments for PKA-thiophosphorylated form of inhibitor-1 were accomplished by using the following heteronuclear 3D spectra: HNCO, HN(CA)CO, HNCA, HN(CO)CA HNCACB and CBCA(CO)NH. All NMR spectra were processed and analyzed using the Bruker TopSpin and Aurelia programs, respectively. ^1^H Chemical shifts were referenced to the ^1^H frequency of the methyl resonances of 3-(trimethylsilyl) propionic acid (TSP) at 0 ppm. The ^15^N and ^13^C chemical shifts were indirectly referenced using the following consensus Ξ ratios of the zero-point frequencies: 0.101329118 for ^15^N/^1^H and 0.251449530 for ^13^C/^1^H. The NMR experiments were performed on Bruker an 800 MHz spectrometer equipped with 5 mm inverse triple-resonance, Z-gradient cryoprobe or a 500 MHz spectrometer equipped with 5 mm inverse triple-resonance, Z-gradient probe. All NMR experiments were performed at 293 K.

### Site-directed mutagenesis

The plasmid, pET11d, encoding the cDNA of human inhibitor-1 was served as a template for site-directed mutagenesis that was carried out by the methods recommended by the manufacturer (Agilent Technologies, Inc., Santa Clara, CA). Phe12 was replaced by Ala by using the primer (5′-CAG CCC CCG AAA GAT CCA GGC CAC GGT CCC GCT GCT GGA GCC-3′). The resulting cDNA of inhibitor-1[F12A] was served as a template for the further site-directed mutagenesis to replace Ile10 by Ala using a primer (5′-GCA AGA CAA CAG CCC CCG AAA GGC CCA GGC CAC GGT CCC GCT GC-3′).

### The inhibitory assay of PP1

PP1 was assayed in presence of various concentrations of PKA-thiophosphorylated form of inhibitor-1 or PKA-thiophosphorylated forms of inhibitor-1[I10A;F12A] using ^32^P-phosphorylase a as a substrate, as described^25^.

## Electronic supplementary material

Supplementary information
