# Supplementary material for: Characterization of the interactions between inhibitor-1 and recombinant PP1 by NMR spectroscopy
Source: Sci Rep. 2018 Jan 8;8:50. doi: 10.1038/s41598-017-18383-x (PMC5758809; doi:10.1038/s41598-017-18383-x)
Supplement: Supplementary file 1 — Supplementary information [file 41598_2017_18383_MOESM1_ESM.pdf]

Supplementary Information

Characterization of the interactions between inhibitor-1 and recombinant PP1 by NMR spectroscopy

Chu-Ting Liang[1,2,†], Yu-Shan Lin[3,†], Yi-Choang Huang[4], Hsien-Lu Huang[5], Jia-Qian Yang[3], Tsung-Hsien Wu[3], Chi-Fon Chang[6], Shing-Jong Huang[7], Hsien-Bin Huang[3,*] and Ta-Hsien Lin[1,2,4,*]

[1]Basic Research Division, Medical Research Department, Taipei Veterans General Hospital, Taipei 11217, Taiwan
[2]Department of Life Sciences and Institute of Genome Sciences, National Yang-Ming University, Taipei 11221, Taiwan
[3]Department of Life Science, National Chung Cheng University, Chia-Yi 62102, Taiwan
[4]Institute of Biochemistry and Molecular Biology, National Yang-Ming University, Taipei 11221, Taiwan
[5]Department of Nutrition and Health Science, Fooyin University, Kaohsiung 83102, Taiwan
[6]Genomics Research Center, Academia Sinica, Taipei 11529, Taiwan
[7]Instrumentation Center, National Taiwan University, Taipei 10617, Taiwan

†The first two authors contributed equally to this work.

*To whom correspondence should be addressed: Hsien-bin Huang, Department of Life Science and Institute of Molecular Biology, National Chung Cheng University, Chia-Yi 62102, Taiwan; Fax:+886-5-2722871; Email: biohbh@ccu.edu.tw and Ta-Hsien Lin, Basic Research Division, Medical Research Department, Taipei Veterans General Hospital, Taipei 11217, Taiwan; Fax: +886-2-8751562; Email: thlin@vghtpe.gov.tw

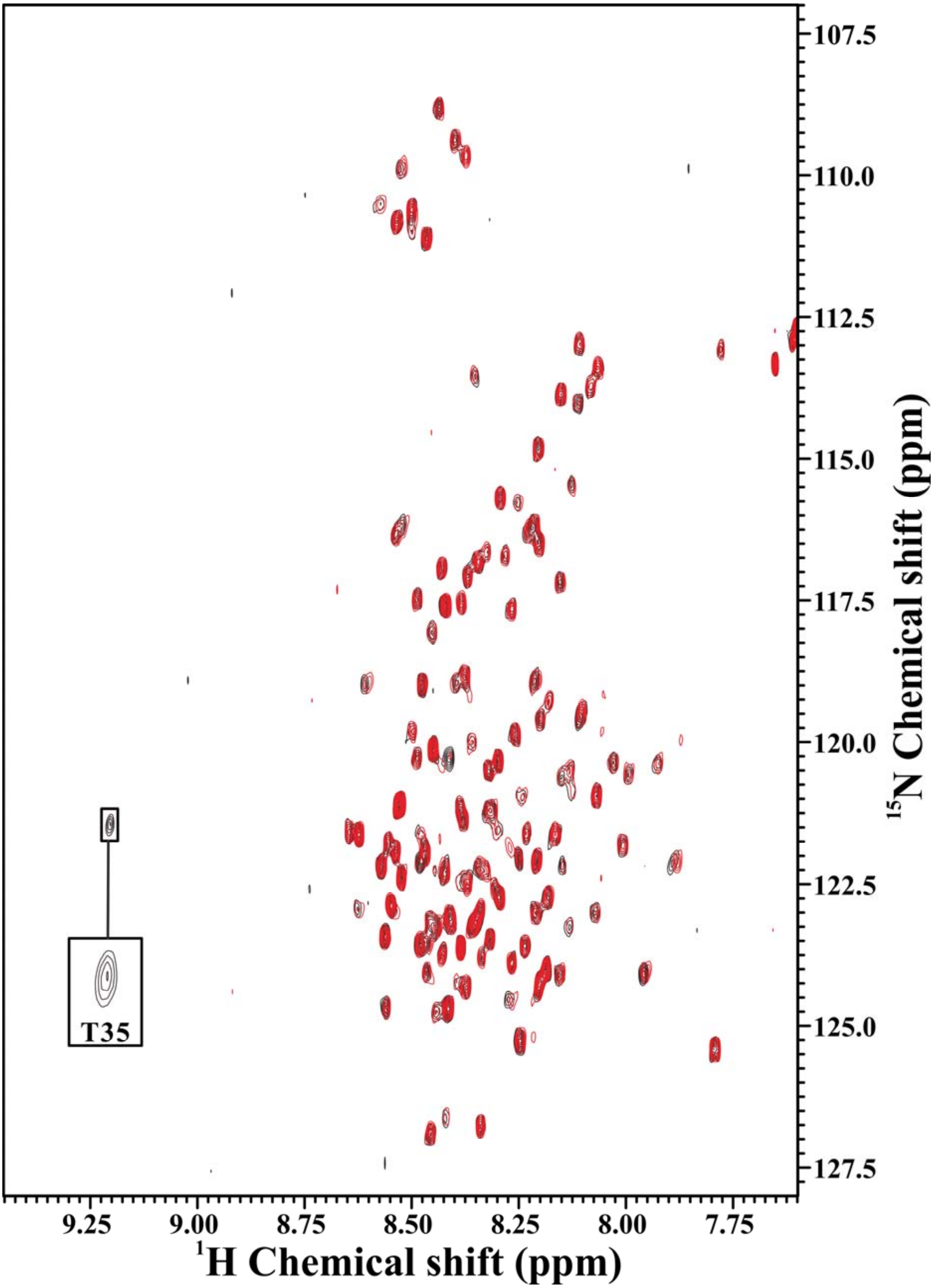

Supplementary Figure 1. Overlay of the two-dimensional ^{1}H-^{15}N-HSQC spectra of PKA-thiophosphorylated form of ^{15}N-labeled inhibitor-1 (40 μM) (red) acquired at 22 minutes after addition of unlabeled recombinant PP1 (8.5 μM). The spectrum acquired without addition of unlabeled recombinant PP1 was colored in black.

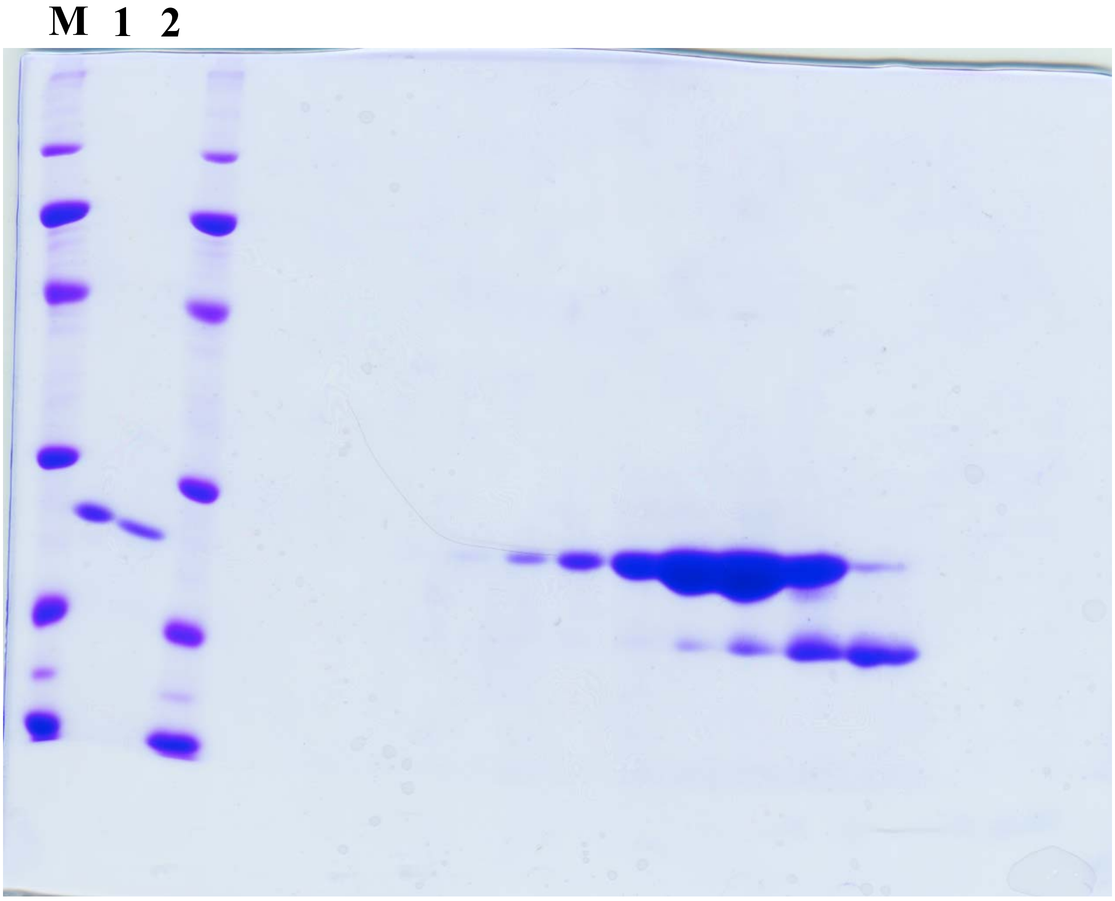

Supplementary Figure 2. Original SDS-PAGE of figure 5. Lane M, 1 and 2 were shown in figure 5.
